# Evaluation of human papillomavirus DNA in colorectal cancer and adjacent mucosal tissue samples

**DOI:** 10.1186/s13027-023-00552-5

**Published:** 2023-11-08

**Authors:** Luisa Galati, Purnima Gupta, Antonio Tufaro, Mariarosaria Marinaro, Concetta Saponaro, David Israel Escobar Marcillo, Donato Loisi, Rajdip Sen, Alexis Robitaille, Rosario N. Brancaccio, Cyrille Cuenin, Sandrine McKay-Chopin, Angelo Virgilio Paradiso, Václav Liška, Pavel Souček, Francesco Alfredo Zito, David J. Hughes, Massimo Tommasino, Tarik Gheit

**Affiliations:** 1 International Agency for Research on Cancer (IARC), 25 Avenue Tony Garnier, CS 90627, 69366 Lyon Cedex 07, France; 2 Institutional BioBank, Istituto Tumori “Giovanni Paolo II”, IRCCS, Bari, Italy; 3 Department of Infectious Diseases, Istituto Superiore di Sanità, Viale Regina Elena 299, Rome, Italy; 4 Pathology Department, IRCCS Istituto Tumori “Giovanni Paolo II”, Bari, Italy; 5 Biomedical Center, Faculty of Medicine in Pilsen, Charles University, Pilsen, Czech Republic; 6 Department of Surgery, Faculty Hospital and Faculty of Medicine in Pilsen, Charles University, Pilsen, Czech Republic; 7 Cancer Biology and Therapeutics Group, School of Biomolecular and Biomedical Science, UCD Conway Institute, University College Dublin, Dublin, Ireland; 8 Present Address: Department of Experimental Oncology, IEO, European Institute of Oncology IRCCS, Milan, Italy; 9 Present Address: Leibniz Institute of Virology, Hamburg, Germany

**Keywords:** Human papillomavirus, HPV16, Colorectal cancer, Next generation sequencing, Luminex

## Abstract

**Background:**

Although the role of viral agents, such as human papillomavirus (e.g. HPV16, HPV18) in colorectal cancer (CRC) has been previously investigated, results remain inconclusive.

**Methods:**

To further evaluate the involvement of oncogenic HPV types in CRC, 40 frozen neoplastic and 40 adjacent colonic tissues collected from Italian patients were analyzed by Luminex-based assays that detect a broad spectrum of HPV types, i.e. Alpha (n = 21), Beta (n = 46) and Gamma HPVs (n = 52). In addition, 125 frozen CRC samples and 70 surrounding mucosal tissues were collected from Czech patients and analyzed by broad spectrum PCR protocols: (i) FAP59/64, (ii) FAPM1 and (iii) CUT combined with Next Generation Sequencing (NGS).

**Results:**

Using Luminex-basedassays, DNA from HPV16 was detected in 5% (2/40) CRC tissues from Italian patients. One HPV16 DNA-positive CRC case was subsequently confirmed positive for E6*I mRNA. Cutaneous beta HPV types were detected in 10% (4/40) adjacent tissues only, namely HPV111 (n = 3) and HPV120 (n = 1), while gamma HPV168 (n = 1) and HPV199 (n = 1) types were detected in adjacent and in tumor tissues, respectively. The NGS analysis of the CRC Czech samples identified HPV sequences from mucosal alpha-3 (HPV89), alpha-7 (HPV18, 39, 68 and 70) and alpha-10 species (HPV11), as well as cutaneous beta-1 (HPV20, 24, 93, 98, 105,124) beta-2 (HPV23), beta-3 (HPV49) and gamma-1 species (HPV205).

**Conclusions:**

Our findings indicate that HPV types belonging to the mucosal alpha, and the ‘cutaneous’ beta and gamma genera can be detected in the colonic mucosal samples with a low prevalence rate and a low number of HPV reads by Luminex and NGS, respectively. However, additional studies are required to corroborate these findings.

## Introduction

Colon rectal cancer (CRC) represents the third most common cancer and is a major cause of mortality worldwide [1]. Tobacco/alcohol consumption, inflammatory syndromes, low fruit/vegetable intake and obesity are associated with an increased risk of CRC [2, 3]. The role of infectious agents, in particular human papillomaviruses (HPVs), has been evaluated in several studies, giving controversial results [4–6]. HPVs are non-enveloped double-stranded DNA viruses that, based on their ability to infect skin or mucosal epithelia, are classified as cutaneous or mucosal [7]. Twelve mucosal alpha HPV types, namely HPV16, 18, 31, 33, 35, 39, 45, 51, 52, 56, 58, and 59, are classified by the International Agency for Research on Cancer (IARC) as high-risk (HR) HPV types (Group 1). Infection with HR HPV types has been associated with human malignancies, i.e. cervical, anogenital and oropharyngeal cancers [6]. Additional alpha HPV types (HPV26, 53, 66, 67, 68, 70, 73 and 82) are considered ‘possible or probable HR’ (Groups 2A and 2B) [6], while HPV6 and HPV11 have been classified as low-risk (LR) types, since they are most frequently detected in benign lesions of anogenital and upper-respiratory tracts [6].

Several case–control studies have shown a higher HPV prevalence, e.g., mucosal types HPV16 and HPV18, in CRC tumor tissue compared to controls [8–10]. A recent meta-analysis supports the role of HPV as a risk factor in CRC development [11]. By contrast, other studies based on the presence of mucosal alpha HPV DNA [12, 13] in tissue or antibodies to HPV16 in blood [14] failed to demonstrate an association between HPV and CRC. Thus, whether HPV (and other infectious agents) contributes to CRC development is still under debate [15, 16].

This study aimed to: (i) characterize the mucosal and cutaneous HPV diversity in CRC using a highly sensitive molecular screening technique (*i.e.,* Luminex-based assay) and next generation sequencing using a relatively large number of CRC specimens collected from Italian (n = 40) and Czech (n = 125) patients, and to; (ii) compare the HPV diversity in CRC to that of adjacent non-malignant mucosa. In addition, the Italian cohort has been fully characterized for HPV DNA and E6*I mRNA to evaluate the role of mucosal HPV in CRC.

## Materials and methods

Frozen colonic samples collected in two different countries, Czech Republic (n = 195 specimens) and Italy (n = 80 specimens), were analyzed for the presence of HPV DNA by NGS or a multiplex Luminex genotyping assay, respectively.

### Italian cohort

#### Study group

CRC frozen tissues (n = 40) and matched surrounding healthy tissues (n = 40) were collected at Istituto Tumori “Giovanni Paolo II” IRCCC Hospital, Bari, Italy between 2017 and 2019. The 40 patients were 26 male and 14 female, with an average age of 67.3. Tumor stage and other baseline characteristics are indicated in Table 1. Data regarding HPV status and cervical lesion history were not available.

**Table 1:** Characteristics of the CRC Italian patients (n = 40)

Characteristics	N (%)
Age at diagnosis, mean (years)	67.3
Sex	
Female	14 (35)
Male	26 (65)
Tumor size (pT)	
pT2	2 (5)
pT3	26 (67)
pT4	11 (28)
Unknown	1
Lymph node metastasis (pN)	
N0	17 (44)
N1	11 (28)
N2	11 (28)
Unknown	1
Distant metastasis (cM)	
M1	9 (60)
Mx	6 (40)
Unknown	25
Grade	
1	7 (19)
2	19 (53)
3	10 (28)
Unknown	4
Tumor localization	
Colon	38 (95)
Rectum	2 (5)
Other	0

#### Nucleic acid extraction

The simultaneous purification of DNA and RNA from frozen tissue was performed at the International Agency for Research on Cancer (IARC, Lyon) using AllPrep DNA/RNA/Protein Mini Kit (Qiagen, Hilden, Germany) following the manufacturer’s instructions. Extracted DNA, RNA and proteins were stored at −80 °C until use.

#### HPV DNA detection assay

The prevalence of a broad spectrum of cutaneous and mucosal HPV types was determined by using type-specific multiplex genotyping (TS-MPG) assays, which is based on multiplex polymerase chain reaction (PCR) and bead-based Luminex technology (Luminex Corp., Austin, TX, USA), as described elsewhere [17]. Briefly, 10 µl of each DNA extract was subjected to a multiplex PCR with HPV type-specific primers targeting a total of 21 mucosal alpha-HPV types, namely HPV6, 11, 16, 18, 26, 31, 33, 35, 39, 45, 51, 52, 53, 56, 58, 59, 66, 68, 70, 73 and HPV82. In addition, multiplex PCRs using type-specific primers were used for the detection of 46 beta-HPV types (HPV5, HPV8, HPV9, HPV12, HPV14, HPV15, HPV17, HPV19, HPV20, HPV21, HPV22, HPV23, HPV24, HPV25, HPV36, HPV37, HPV38, HPV47, HPV49, HPV75, HPV76, HPV80, HPV92, HPV93, HPV96, HPV98, HPV99, HPV100, HPV104, HPV105, HPV107, HPV110, HPV111, HPV113, HPV115, HPV118, HPV120, HPV122, HPV124, HPV143, HPV145, HPV150, HPV151, HPV152, HPV159 and HPV174), and 52 gamma-HPVs (HPV4, HPV48, HPV50, HPV60, HPV65, HPV88, HPV95, HPV101, HPV103, HPV108, HPV109, HPV112, HPV116, HPV119, HPV121, HPV123, HPV126, HPV127, HPV128, HPV129, HPV130, HPV131, HPV132, HPV133, HPV134, HPV148, HPV149, HPV156, HPV161, HPV162, HPV163, HPV164, HPV165, HPV166, HPV167, HPV168, HPV169, HPV170, HPV171, HPV172, HPV173, HPV175, HPV178, HPV179, HPV180, HPV184, HPV197, HPV199, HPV200, HPV201, HPV202 and SD2). Two primers for beta-globin were also used to assess the quality of the extracted DNA. After PCR amplification, 10 µl of each reaction was analysed using a multiplex Luminex-based assay as detailed previously [17].

#### E6*I mRNA analysis

RT-PCR was carried out using the QuantiTect Virus Kit (Qiagen, Hilden, Germany), in a total volume of 25 μl containing 5 μl of 5 × QuantiTect Virus Mastermix, 0.25 μl of 100 × QuantiTect Virus RT Mix, 0.4 μM of each oligonucleotide, and 1 μl RNA as described previously [18]. HPV specific primers and probes from a HPV type-specific E6*I mRNA assay [18] were used for the detection of viral transcripts. The assay amplifies a 65–75 base pair amplicon of HPV and an 81 base pair amplicon of ubiquitin C (ubC) cDNA. Biotinylated amplification products were hybridized to ubC and HPV type-specific probes, representing splice junction sequences, on Luminex beads, followed by staining with streptavidin–phycoerythrin, and quantified in a Luminex analyzer as previously described by Halec et al. 2013 [18].

#### HPV DNA detection by in situ hybridization (ISH)

The 3-µm thick sections of formalin-fixed, paraffin-embedded (FFPE) tissue were tested by in situ hybridization (*ISH)* using the HPV Probe (types 16, 18, 31, 33, 51) [HPV Probe Leica Biosystems, Leica, Newcastle, UK. Catalog No: PB0829] for the qualitative detection of HPV DNA, on the automated Leica BOND‐III system, according to the manufacturer’s instructions.

Briefly, slides underwent deparaffinization with the Bond Dewax solution followed by epitope retrieval using Stringency Wash solution and addition of biotinylated HPV-type-specific DNA probes followed by anti-Biotin antibody. After washings, post primary and polymer incubation, DAB staining and Hematoxylin counterstain, were performed. Slides were analyzed by using an Axio Imager A1 (Zeiss, Göttingen Germany).

### Czech cohort

#### Study group

The CRC patients were recruited from the Department of Surgery, Teaching Hospital and Medical School, in Pilsen, Czech Republic, between January 2008 and November 2011.

Collection, processing of tissues and data acquisition of the Czech samples, was performed as previously described [19]. The clinical data, including age at diagnosis, sex, pTNM (Tumor stage, Regional lymph node involvement and distant metastasis) staging, histological grade of the tumor, and primary tumor localization were obtained from patient’s medical records (Table 2). After surgical resection, colorectal tumor and adjacent non-malignant tissues were snap frozen and stored at -80 °C.

**Table 2:** Clinical characteristics of the Czech patients (*n* = 125)

Characteristics	N (%)
Age at diagnosis, mean (years)	69
Sex	
Female	37 (29.6)
Male	88 (70.4)
Tumor size (pT)	
pT2	7 (5.6)
pT3	105 (84)
pT4	13 (10.4)
Lymph node metastasis (pN)	
pN0	73 (58.4)
pN1	36 (28.8)
pN2	16 (12.8)
Distant metastasis (cM)	
cM0	115 (92)
cM1	10 (8)
Pathological grade (G)	
G1	16 (12.8)
G2	90 (72)
G3	17 (13.6)
Gx	2 (1.6)
Tumor localization	
Colon	84 (67.2)
Rectum	34 (27.2)
Other^a^	7 (5.6)

^a^Six cases were C18/C18.4/C18.5 with subtotal colectomy surgical procedure and the site of primary was not specified while one patient was C18.5 (classifications according to the 10th revision of the International Statistical Classification of Diseases, Injury and Causes of Death)

#### DNA extraction and PCR amplification

Total DNA was extracted from frozen CRC (n = 125), and available surrounding tissues (n = 70) using the DNeasy Blood and Tissue Kit following the manufacturer’s instructions (Qiagen, Courtaboeuf, France). DNA samples were sent to IARC for HPV DNA characterization by PCRs combined with NGS. Total extracted DNA was amplified using three degenerated primer sets (i) FAP59/64 (FAP), (ii) FAPM1 and* (*iii) CUT protocols as previously reported [20–22].

#### Library preparation and NGS assay

PCR amplicons of the expected size (about 480 bp) were generated from 41 and 78 CRC samples using FAP and FAPM1 primers, respectively. In adjacent CRC specimens, PCR amplicons were generated from 16 and 45 specimens using FAP and FAPM1 primers, respectively. Finally, amplicons from 98 CRC and 37 adjacent samples were obtained using CUT primers and. PCR amplicons of the expected size were purified as previously described [21] and divided by PCR protocols (FAP, FAPM1, CUT) and tissue specimens (CRC and adjacent CRC) in a total of 6 different pools using the same volume of each purified amplicon. NGS analysis was performed on the pooled amplicon-based library (Nextera DNA Flex) using the Illumina MiSeq sequencer (2 X 150 paired-end reads, MiSeq reagent kit v3) (Illumina, San Diego, CA, USA) as previously reported [21]. Bioinformatic analyses were performed using PVAmpliconFinder tool [23], and all the results were based on the homology-based classification using the evolutionary placement algorithm in RAxML (Randomized Axelerated Maximum Likelihood) [24].

## Results

### Italian cohort

HPV prevalence was determined in a series of frozen surrounding (n = 40) and CRC matched tissues (n = 40) from Italy, using Luminex-beads based assays. DNA from HPV16 was found in two CRC tissue samples (2/40, 5.0%) from male patients, and in one adjacent tissue (Table 3). One of the HPV16 DNA-positive CRC tissues was also positive for HPV16 E6*I mRNA by RT-PCR. In addition, all HPV DNA positive cases (n = 3) were negative in FFPE tissue samples by HPV-in situ hybridization (HPV *ISH*) (Data not shown).

**Table 3:** Distribution of Human papillomaviruses in CRC (n = 40) and adjacent (n = 40) tissues

Species	HPV type	Adjacent CRC	CRC	Total
		*n* (%)	*n* (%)	*n* (%)
Alpha-9	HPV16	1 (2.5%)	2 (5%)	3 (3.7%)
Beta-2	HPV111	3 (7.5%)	–	3 (3.7%)
Beta-2	HPV120	1 (2.5%)	–	1 (1.2%)
Gamma-8	HPV168	1 (2.5%)	–	1 (1.2%)
Gamma-12	HPV199	–	1 (2.5%)	1 (1.2%)

Cutaneous beta HPV111 (n = 3) and HPV120 (n = 1) DNA was detected in 10% (4/40) of surrounding tissue samples. Cutaneous gamma HPV DNA from HPV168 and HPV199 types was found in adjacent and tumor tissues, respectively (Table 3). Altogether, a total of 5 different HPV types were detected in colorectal tissues, as shown in Table 3.

### Czech cohort

A broad-spectrum PCR combined with NGS was employed to detect HPV DNA in CRC samples from the Czech Republic. A total of 18,012,835 raw reads were generated of which the majority (99.9%) of reads align to the host human genome, while 18,893 reads (0.1%) were classified into the *Papillomaviride* family, as reported in Table 4. Almost all (99.9%) of the papillomavirus reads were generated by the FAP PCR protocols. Specifically, 7,338 reads (38.8%) and 10,892 reads (57.7%) were generated by FAP59/64 primers in CRC and adjacent non-neoplastic CRC samples, respectively. A total of 3.4% (652 reads) were generated by FAPM1 primers in adjacent non-neoplastic specimens.

**Table 4:** HPV sequences in CRC and adjacent tissues according to PCR protocols and NGS analysis

Species	HPV type	Adjacent CRC			CRC		
		Number of reads					
		PCR protocol FAP59/64	PCR protocol FAPM1	PCR protocol CUT	PCR protocol FAP59/64	PCR protocol FAPM1	PCR protocol CUT
Alpha-3	HPV89	–	–	2	–	–	–
Alpha-7	HPV18	2	–	–	–	–	–
Alpha-7	HPV39	–	–	2	–	–	–
Alpha-7	HPV68	–	–	–	1815	–	–
Alpha-7	HPV70	–	391	–	–	–	–
Alpha-10	HPV11	–	–	–	–	–	2
Beta-HPV	HPV-mm292c14	876	–	–	–	–	–
Beta-1	HPV105	–	–	–	–	–	2
Beta-1	HPV124	–	89	–	–	–	–
Beta-1	HPV20	–	–	–	647	–	–
Beta-1	HPV24	–	–	–	3843	–	–
Beta-1	HPV93	10,014	–	–	–	–	–
Beta-1	HPV98	–	–	–	39	–	–
Beta-2	HPV23	–	8	–	–	–	–
Beta-3	HPV49	–	107	–	–	–	–
Gamma-HPV	HPV-mDysk3	–	–	2	–	–	–
Gamma-HPV	HPV-mSK245	–	–	–	995	–	–
Gamma-HPV	HPV205	–	57	–	–	–	–

PCR protocols generated a total of 2214 reads related to alpha HPVs, of which 1,815 reads, all assigned to HPV68, were found in tumor samples, while 391 reads assigned to HPV70 were found in adjacent tissue (Table 4). However, a very few sequences related to HPV18 and 39 belonging to α-7 species were detected in healthy surrounding tissues, as well as HPV89 from α-3 species (Table 4).

A total of 15,625 reads related to beta HPVs were identified, of which 4,529 reads from HPV20, HPV24, HPV98 and HPV105, all from beta-1 species, were located in CRC samples, while the majority, i.e., 11,094 reads from beta-2 HPV23, beta-3 HPV49, beta-1 HPV93, beta-1 HPV124 and unreferenced HPV-mm292c14, were found in non-neoplastic tissues.

Only 1,054 reads belonged to the genus gamma, with reads assigned to HPV-mSK245 (n = 995) and gamma-1 HPV205 (n = 57) and HPV-mDysk3 (n = 2) observed in tumor and non-neoplastic tissues, respectively. Altogether, a total of 18 different HPV sequences were detected in colorectal tissues, although some of these had very low read numbers (Table 4).

## Discussion

In the present study, we determined the presence of a broad spectrum of mucosal and cutaneous HPV types in frozen CRC tissue samples using several PCR protocols and molecular assays [17, 20–22]. Using a Luminex-based assay, only a small fraction of samples tested positive for mucosal HPVs as indicated by the presence of HPV16 DNA in 2 out 40 CRC cases (5%) *vs.* 1 out 40 adjacent tissues. Moreover, HPV16 DNA positivity was confirmed to be transcriptionally active by the presence of HPV16 E6*I mRNA in one of the HPV16 DNA-positive CRC tissues. However, it is possible that this CRC with HPV E6 mRNA expression was not a primary cancer as the possibility of metastasis of HPV-related squamous neoplasia located at a different anatomical site cannot be excluded. The HPV *ISH* analysis did not confirm the presence of HPV16 in both HPV16 DNA-positive cases. This result can be explained by a lack of analytical sensitivity of the *ISH* technique compared to the Luminex-based assay in detecting viral DNA.

Next, a broad spectrum NGS based assay was used to determine the presence of HPV in a large Czech sample collection. As per NGS analysis, reads from HPV18 and two related types HPV70 and HPV68 of mucosal alpha-7 species were found in colonic tissues. In contrast to Italian cases, HPV16 was not detected in any of the NGS pools. However, this result may be due either to the absence of HPV16 in Czech colon specimens or linked to the methodology based on the use of degenerated primers (NGS) versus specific primers and probes which constitute the backbone of the Luminex-based assays. Interestingly, the presence of alpha-7 HPV in the colonic mucosa has been reported by other studies using different strategies. The presence of oncogenic HPV18 was previously reported in CRC [25] as well as in healthy mucosal gut [26] and anorectum [27]. In addition, HPV68 was previously reported in human gut specimens from healthy individuals [26]. However, the presence of HPV18 in CRC cases is supported by a small number of reads which may reflect a low viral load. Alternatively, this result can also be explained by a lack of specificity of the amplification due to the use of degenerated primers.

We also detected DNA sequences from several beta HPV types from species beta-1, beta-2 and beta-3 (HPV20, 23, 24, 49, 93, 98, 124) and from gamma-1 (HPV205) (Tables 3, 4). Cutaneous beta HPV types, specifically beta-2 HPV111 (n = 3) and HPV120 (n = 1), were only detected by Luminex assay in DNA extracted from surrounding tissues. Epidemiological studies have reported that beta-1 and beta-2 species are mainly observed in the skin but are also present in various mucosal sites [28]. Regarding gamma HPV types, only 3 HPV sequences (HPV205 and two unclassified types) were identified by NGS, while the two gamma types HPV168 and HPV199 were detected by Luminex assay. Several gamma HPVs have been previously isolated from various mucosal sites such like nasopharynx, oral cavity, anal mucosa and cervical swabs, as reviewed in [28].

Using different methodologies for detecting HPV DNA, the current study provided evidence for a low HPV prevalence in Italian CRC specimens, and a low number of NGS HPV reads in the Czech cohort, in agreement with previous studies [12, 29–32]. It is noteworthy that overall few HPV types were found (9 HPV types in CRC vs. 15 HPV types in adjacent tissues), despite the use of two different methodologies and two different cohorts. Overall, HPV types belonging to the alpha, beta and gamma genus were detected by both molecular techniques. The distribution of HPV types was different between Italian and Czech CRC samples which can be explained by (i), the use of different methodologies that doesn’t allow for inter-cohort comparisons, and (ii), the geographical origin of the specimens. While Luminex-based assays allow for highly sensitive and specific detection of a restricted number of HPV types, as described in Schmitt et al. [17], the second approach based on the use of degenerated primers combined with next-generation sequencing allows for a larger and broader detection of HPV types, including unknown HPV types, as reported in Brancaccio et al. [21]. Furthermore, specific HPV-types could be over and/or underrepresented due to the use of degenerated primers, leading to over and/or under detection of some HPV-types by NGS. Moreover, the pool-based strategy applied for NGS protocol constitutes a limitation in this study as it does not allow for HPV prevalence determination. In addition, while both DNA and RNA samples could be generated from the Italian samples, only DNA samples were available for the Czech cohort which constitutes another limitation in the current study.

However, the strength of this study is the use of both a highly sensitive molecular screening technique and a deep sequencing for a broad spectrum of cutaneous and mucosal HPV types in a substantial number of CRC and adjacent tissue samples.

In agreement with a previous study conducted on healthy individuals [26], we detected HPV DNA from few alpha, beta and gamma HPV types in the adjacent non-malignant colonic mucosa, although we cannot exclude that the viral DNA was just a passenger in the colon while the original infection occurred at other anatomical sites (e.g. digestive tract).

## Conclusions

This study investigated the presence of a broad spectrum of HPV types, some of which are oncogenic, in the colonic mucosa using validated Luminex and NGS DNA assays. The low prevalence of HPV types by Luminex and the low number of HPV reads by NGS do not support an appreciable role of HPVs in CRC development. However, since an etiological role of infectious agents in CRC cannot be completely excluded, further and larger epidemiological investigations in diverse settings are needed to evaluate the impact of HPV and other oncogenic viruses in CRC samples.

## Data Availability

The datasets analysed during the current study are available from the corresponding author on reasonable request.
